# Involvement of the Cdc42 Pathway in CFTR Post-Translational Turnover and in Its Plasma Membrane Stability in Airway Epithelial Cells

**DOI:** 10.1371/journal.pone.0118943

**Published:** 2015-03-13

**Authors:** Romain Ferru-Clément, Fleur Fresquet, Caroline Norez, Thierry Métayé, Frédéric Becq, Alain Kitzis, Vincent Thoreau

**Affiliations:** 1 Laboratoire Génétique des Maladies Rares, Université de Poitiers, Poitiers, France; 2 Service de Génétique, Centre Hospitalier Universitaire de Poitiers, Poitiers, France; 3 Laboratoire Signalisation et Transports Ioniques Membranaires, CNRS, Université de Poitiers, Poitiers, France; 4 Laboratoire de Biophysique Médicale, Centre Hospitalier Universitaire de Poitiers, Poitiers, France; NHLBI, NIH, UNITED STATES

## Abstract

Cystic fibrosis transmembrane conductance regulator (CFTR) is a chloride channel that is expressed on the apical plasma membrane (PM) of epithelial cells. The most common deleterious allele encodes a trafficking-defective mutant protein undergoing endoplasmic reticulum-associated degradation (ERAD) and presenting lower PM stability. In this study, we investigated the involvement of the Cdc42 pathway in CFTR turnover and trafficking in a human bronchiolar epithelial cell line (CFBE41o-) expressing wild-type CFTR. Cdc42 is a small GTPase of the Rho family that fulfils numerous cell functions, one of which is endocytosis and recycling process *via* actin cytoskeleton remodelling. When we treated cells with chemical inhibitors such as ML141 against Cdc42 and wiskostatin against the downstream effector N-WASP, we observed that CFTR channel activity was inhibited, in correlation with a decrease in CFTR amount at the cell surface and an increase in dynamin-dependent CFTR endocytosis. Anchoring of CFTR to the cortical cytoskeleton was then presumably impaired by actin disorganization. When we performed siRNA-mediated depletion of Cdc42, actin polymerization was not impacted, but we observed actin-independent consequences upon CFTR. Total and PM CFTR amounts were increased, resulting in greater activation of CFTR. Pulse-chase experiments showed that while CFTR degradation was slowed, CFTR maturation through the Golgi apparatus remained unaffected. In addition, we observed increased stability of CFTR in PM and reduction of its endocytosis. This study highlights the involvement of the Cdc42 pathway at several levels of CFTR biogenesis and trafficking: (i) Cdc42 is implicated in the first steps of CFTR biosynthesis and processing; (ii) it contributes to the stability of CFTR in PM *via* its anchoring to cortical actin; (iii) it promotes CFTR endocytosis and presumably its sorting toward lysosomal degradation.

## Introduction

Cystic fibrosis (CF) is the most common recessive inherited disorder in Caucasian populations. It is caused by mutations in the gene encoding the CF transmembrane conductance regulator (CFTR) [1,2]. This protein is a member of the adenosine triphosphate (ATP)-binding cassette (ABC) transporter family. After maturation throughout the endoplasmic reticulum and the Golgi complex, it is addressed to the apical membrane of epithelial cells [3,4], where it functions as a chloride channel. In addition, it has been shown that plasma membrane-resident CFTR (PM-CFTR) undergoes rapid cycles of endocytosis mediated by clathrin-coated vesicles, followed by recycling to the plasma membrane (PM) [5]. The CFTR mutation F508del accounts for 70–75% of the deleterious alleles found amongst CF patients. *Per se*, this protein mutation does not preclude CFTR chloride channel gating [6]. However, as folding of the newly synthesized protein in the endoplasmic reticulum (ER) is unsuccessful, it is fully degraded by the ubiquitin-proteasome pathway before reaching the Golgi complex [7,8]. Subsequently, it has been shown in CFTR-transfected BHK cells that endocytosis rates are identical for WT and rescued (r)F508del-CFTR, whereas recycling to PM is less efficient for the mutant [9]. However, in polarized airway epithelial cells, accelerated endocytosis of rF508del-CFTR is apparently primarily involved [10]. Moreover, unfolded rF508del-CFTR at the cell surface appears tagged by a peripheral ubiquitination mechanism for targeting to lysosomal degradation [11]. Because of these dysfunctions of the most common mutant, the trafficking mechanisms of CFTR to and from PM (initial targeting, internalization, recycling) have been the subject over the years of considerable attention.

Numerous protein actors have been shown to belong to CFTR interactome and/or to accompany CFTR intracellular routing and channel activity [12–14]. In this study, we focused on the impact of the Cdc42-dependent pathway on CFTR trafficking in epithelial cells. Cdc42 is a member of the Rho family of small GTPases, of which the role in actin cytoskeleton remodelling has been extensively characterized over recent decades [15]. Amongst the plethora of cell functions in which these proteins are implicated, it has been shown in several cell types that Cdc42 can regulate the endocytosis and/or recycling of some plasma membrane proteins [16,17]. GTP-bound Cdc42 binds to and activates downstream effectors such as proteins of the WASP (Wiskott Aldrich syndrome protein) and Toca (transducer of Cdc42-dependent actin assembly) families, which in turn cooperate as they trigger the branching of actin filaments *via* the Arp2/3 complex. Actin cytoskeleton then contributes to membrane curvature followed by vesicle scission and forms the track along which vesicles move throughout peripheral areas of the cytosol.

As a cell-permeant pharmacological inhibitor of N-WASP, wiskostatin has been reported to elicit inhibition of CFTR-mediated chloride currents and reduction of cell surface CFTR protein [18]. Following biotinylation-based experiments carried out in CFTR-transfected BHK cells, the authors concluded that due to wiskostatin treatment, CFTR endocytosis is initially increased. As CFTR appears to accumulate in intracellular compartments, its recycling back to PM seems to be hampered over longer time periods. As N-WASP, an ubiquitous downstream effector of Cdc42 involved in regulation of actin organization, appears to be involved in CFTR trafficking, the Cdc42 pathway of endocytosis and recycling is a possible component of CFTR transport machinery.

In this paper, we studied the relevance of this hypothesis in a human bronchiolar epithelial cell line, CFBE41o- (established from a CF patient homozygous for the F508del mutation) overexpressing wild-type CFTR (CFBE-wtCFTR) [19]. Firstly, we treated cells with two pharmacological compounds: wiskostatin, which has been used to impact CFTR trafficking, and ML141, a newly characterized specific inhibitor of Cdc42 protein [20,21]. Secondly, the Cdc42 pathway was impaired by RNA interference against Cdc42 or N-WASP. Our data demonstrate that the Cdc42 pathway is involved in control of total CFTR amount within the cell, as well as in the PM-stability of CFTR *via* its anchoring to the cortical actin cytoskeleton and/or *via* regulation of its endocytosis.

## Materials and Methods

### Cell culture

The experiments were performed with the previously used and acknowledged human bronchiolar epithelial cell line CFBE41o- overexpressing wild-type CFTR (CFBE-wtCFTR) [19], a generous gift from John Paul Clancy (University of Alabama at Birmingham, USA). Cells were cultured at 37°C in 5% CO_2_ in αMEM medium with Glutamax-I (Life Technologies) supplemented with 10% fetal calf serum (Eurobio, France), 100 IU/mL penicillin (Panpharma SA, France), 100 μg/mL streptomycin (Panpharma SA, France) and under 0.5 μg/mL puromycin (InvivoGen) selection.

### RNA interference

siRNA specifically targeting human genes (sense strand sequences indicated in S1 Table) and Universal Negative Control siRNA were purchased from Eurogentec (Seraing, Belgium). Cells were transfected using Lipofectamine 2000 reagent (Invitrogen) according to the manufacturer’s protocol. Depletions were assessed by real-time RT-PCR (primer sequences available upon request) and/or western blot 48 h after transfection.

### Antibodies and chemicals

The following primary antibodies were used in this study: mouse monoclonal antibodies anti- CFTR (clone M3A7, Millipore, for immunoblot, and clone 24–1, R&D Systems, for immunoprecipitation), anti-Na^+^/K^+^ ATPase α1 subunit (clone C464.6, Santa Cruz Biotechnology), anti-ezrin (BD Transduction Laboratories), anti-Cdc42 (BD Transduction Laboratories), anti-ZO-1 (Invitrogen), anti β-actin (clone AC-74, Sigma-Aldrich), anti-Rac1 (BD Transduction Laboratories), anti-RhoA (clone 26C4, Santa Cruz Biotechnology); rabbit polyclonal antibodies anti dynamin 2 (Abcam), anti-caveolin 1 (Synaptic Systems, Germany) and rabbit monoclonal antibody anti-N-WASP (clone 30D10, Cell Signalling). Peroxydase-linked secondary antibodies for Western blot were obtained from Sigma-Aldrich: goat anti-mouse IgG (Fc specific) and goat anti-rabbit IgG (whole molecule). For fluorescent imaging, FITC-conjugated goat anti-mouse IgG (H+L) (Jackson ImmunoResearch Laboratories), phalloidin-TRITC (Sigma Aldrich) and TO-PRO-3 iodide (Invitrogen) were used. ML141, a selective inhibitor of Cdc42 GTPase [20], was a generous gift from Jennifer Golden (Kansas University Specialized Chemistry Center, USA). Unless otherwise indicated, all other chemicals were purchased from Sigma-Aldrich.

### Immunoblot

Cells were solubilized in lysis buffer: 10 mM Tris-HCl (pH 7.5), 1% Nonidet P-40, 0.5% deoxycholate supplemented with a protease inhibitor cocktail (Roche Applied Science) and 2 mM AEBSF. Cell lysate homogenization, SDS-PAGE, and Western blot were performed as previously described [22]. Bands were quantified by densitometry using Scion Image software. To compare sets of data, we used Dunnett’s Multiple Comparison Test. Each result reported in this paper is representative of at least 3 independent experiments.

### Biochemical determination of CFTR amount at the cell surface

Cells were washed three times with PBS (phosphate buffered saline, pH 8) containing 1 mM MgCl_2_ and 0.1 mM CaCl_2_ (PBS-CM8) at 4°C, and maintained on ice to block protein trafficking. Cell surface-exposed proteins were labelled at 4°C for 25 min using EZ-Link Sulfo-NHS-SS-Biotin (Thermo Scientific) at 0.5 mg/mL in PBS-CM8. Reaction was blocked through three 5 min incubations with Quenching Buffer (PBS-CM8 supplemented with 0.5% BSA and 50 mM glycine). After three PBS washes, cells were lysed as described above. High Capacity Streptavidin Agarose resin (Thermo Scientific) was added to 100 μg of protein from cell lysate and labelled protein binding was allowed to occur overnight, under gentle shaking, at 4°C. Bound complexes were washed three times with NET buffer: 50 mM Tris-HCl (pH 7.4), 150 mM NaCl, 0.05% Nonidet P-40, 5 mM EDTA. Finally, labelled proteins were eluted by denaturation with Laëmmli’s buffer for 60 min at 37°C prior to Western blot analysis. Whenever required, this protocol was modified as explained in the text and in figures.

### Endocytic assay

Relative endocytic rates were evaluated by quantifying the initially surface-exposed CFTR amount, which was internalized in 5 min. After surface biotinylation, plasma membrane proteins were allowed to enter the inner cell compartment by 5 min incubation with αMEM at 37°C. The labelling agent was then cleaved from proteins that remained surface-exposed by six rounds of disulfide bond reduction, for 20 min each at 4°C in Stripping Buffer: 50 mM Tris-HCl (pH 8.6), 100 mM NaCl, 1 mM EDTA, 0.2% BSA, 100 mM sodium 2-mercaptoethanolsulfonate. Reaction was blocked through three 5 min incubations with Quenching Buffer. Finally, the cells were washed three times with PBS and reduction-protected (*i*.*e*. internalized) labelled proteins were extracted from at least 500 μg of cell lysates. Stripping efficiency was controlled without incubation at 37°C, resulting in a signal loss of the initial surface-exposed CFTR over 95% (data not shown). Endocytic rates were expressed as a ratio of internalized CFTR to initially surface-exposed CFTR. Whenever required, this protocol was modified as explained in the text and in figures.

### GTPase activation assay

Pull-down assays were performed using fusion proteins GST-Rhotekin-Rho-binding-domain (resp. GST-PAK-CRIB-domain) as baits to quantify GTP-bound RhoA protein (resp. Rac1 or Cdc42 proteins). Recombinant fusion proteins were produced in *E*. *coli* and purified with Glutathione Sepharose High Performance beads (GE Healthcare) as previously described [22]. After treatments, CFBE41o- cells were lysed in LIL buffer: 25 mM Tris-HCl (pH 7.5), 150 mM NaCl, 5 mM MgCl_2_, 1% NP-40, 1 mM DTT, 5% glycerol, supplemented with a protease inhibitor cocktail. In the clarified lysates, GTPases were then loaded with GTP-γS by first adding 10 mM EDTA, then 0.1 mM GTP-γS, and incubating for 15 min at 30°C. After 60 mM MgCl_2_ addition, pull-downs were performed and analyzed by Western blot as previously described [22]. Results are calculated as the ratio of activated to total GTPase.

### Immunostaining and confocal microscopy

Cells were seeded at 75,000 cells per cm^2^ density on 0.4 μm pore size Transwell permeable support (Costar) and were grown until a monolayer was established. They were then fixed in 3.7% paraformaldehyde and permeabilized in PBS containing 0.5% Triton X-100. Unspecific antigen sites were blocked with PBS containing 0.5% bovine serum albumin. Cells were incubated with anti ZO-1 antibody (1:100), overnight at 4°C. Cells were then incubated with secondary antibodies (1:100), 0.5 mg/mL phalloidin-TRITC and TO-PRO-3 (Invitrogen, 1:500) for 1 hour at room temperature. Inserts were mounted with Mowiol mounting medium. Fluorescence was examined with a spectral confocal, station FV-1000 installed on an inverted microscope IX-81 (Olympus, Tokyo, Japan). Z-series were obtained using a 0.15 μm step.

### Actin polymerization assay

Cells grown to confluence in 6-well plates were fixed for 15 min in 3.7% paraformaldehyde in PBS-CM8 and permeabilized for 15 min with 0.1% Triton X-100 in PBS-CM8. They were then incubated for 1 h with 0.25 μM phalloidin-TRITC in a buffer containing 20 mM KH_2_PO_4_, 2 mM MgCl_2_, 5 mM EGTA, 10 mM PIPES (pH 6.8 with KOH). After rinsing, bound phalloidin was extracted overnight with methanol at 4°C. The ethanol extracts were dispatched in three wells of a 96-well microplate and fluorescence was measured with a Mithras LB940 (Berthold) reader. Emission measurements were normalized to protein levels.

### Iodide efflux

The gating of CFTR Cl^-^ channels was assessed by the iodide (^125^I^-^) efflux technique as already described [23]. Curves were constructed by plotting the rate of ^125^I^-^ versus time. All comparisons were based on maximal values for the time-dependent rates (k = peak rates, min^-1^), excluding the points used to establish the baseline (k_peak_—k_basal_, min^-1^). Result significances were calculated by two-tailed *t* test or ANOVA test, using GraphPad Prism 6 software. Forskolin and genistein were purchased from LC Laboratories, USA and diluted in DMSO.

### Pulse-chase analysis

For metabolic labelling experiments, subconfluent cells grown in 60 mm dishes were transfected with siRNA and cultured for 48 h. The cells were then starved for 30 min in methionine- and cysteine-free MEM (Life Technologies) before being incubated for 15 min in the same medium, supplemented with 200 μCi/mL of EasyTag EXPRESS^35^S Protein Labeling Mix (Perkin Elmer). For the chase, the labelling medium was replaced by regular medium and the cells were homogenized as described above after chase (0, 0.5, 1, 2 and 4 h). Radiolabelled CFTR was then isolated by immunoprecipitation: equal amounts of proteins were incubated overnight at 4°C with CFTR monoclonal antibody and 30 min at 4°C with Protein G Sepharose 4 Fast Flow (GE Healthcare). Bead-bound complexes were washed, denatured in Laëmmli’s buffer for 60 min at 37°C and separated on 5% SDS-PAGE. For autoradiography, the gels were dried and exposed to Hyperfilm ECL (GE Healthcare) at -80° for up to 30 days. Bands were quantified by densitometry using Scion Image software.

## Results

### Pharmacological inhibitors of Cdc42 pathway decrease plasma membrane CFTR amount and CFTR activity

**Fig 1:**
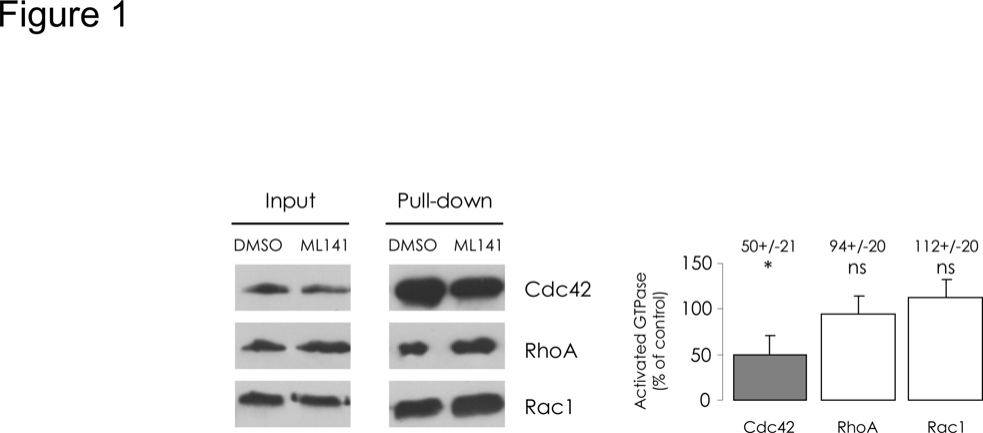
ML141 selectively reduces the GTP-bound form of Cdc42. Before lysis, cells were submitted to 10 μM ML141 for 30 min and 1% DMSO (v/v) was used as the control condition. After enrichment of the activated forms of GTPases in the clarified lysates, GST-pull-down was performed. Cdc42, Rac1 or RhoA protein amounts were then assessed in the resulting samples. (A) Representative Western blot images are shown. Densitometric quantification of bands was normalized to DMSO condition. (B) Histogram displays relative activated GTPase amounts, expressed as the percentage of control. Data represent means ± SEM of 3 independent experiments each performed in duplicate. *: p<0.05, ns: non significant.

Few compounds specifically interfering with Cdc42 pathway are available. To inhibit Cdc42, we used ML141, a new lead molecule that was identified by the Kansas University Specialized Chemistry Center [20]. First, we tested its inhibition selectivity amongst several GTPases, before using it to study CFTR trafficking. We performed an *in vitro* assay based on the binding of GTPases to an interaction domain of their effectors, to estimate the activation state of RhoA, Rac1 and Cdc42. CFBE-wtCFTR cells were treated with 10 μM ML141 for 30 min, according to the manufacturer’s suggestion, and the ratios of activated to total GTPases in the cells were determined (Fig. 1). As expected, when the cells were exposed to ML141 the proportion of activated Cdc42 was reduced to 50%, compared to the DMSO control. In the cases of Rac1 and RhoA, we found no impact of ML141. As its inhibitory effect appeared specific in CFBE-wtCFTR cells, in the subsequent steps of our study we exposed the cells for 30 min to 10 μM ML141.

Wiskostatin, an inhibitor of N-WASP, had previously been used to study CFTR trafficking; in CFTR-transfected BHK cells, the authors observed a decrease of PM-CFTR amount upon wiskostatin treatment [18]. We consequently investigated the effect of this drug in the epithelial cell model that we chose to use for this study. In the previous work, the authors exposed the cells to 100 μM wiskostatin during 2 h for the experiments in which they estimated the amount of PM-CFTR and the CFTR internalization rate. In our experiments, 100 μM wiskostatin treatment appeared harmful for CFBE-wtCFTR cells; lower adherence to the culture vessels led to substantial cell loss during the numerous washing steps of the biochemical assays, which deprived the results of significance. To establish convenient treatment conditions, we performed PM-CFTR quantification in the presence of wiskostatin (S1 Fig.); to obtain results similar to those achieved after 30 min of incubation with 100 μM wiskostatin, we treated the cells for 120 min with 10 μM wiskostatin, without their undergoing any apparent damage.

**Fig 2:**
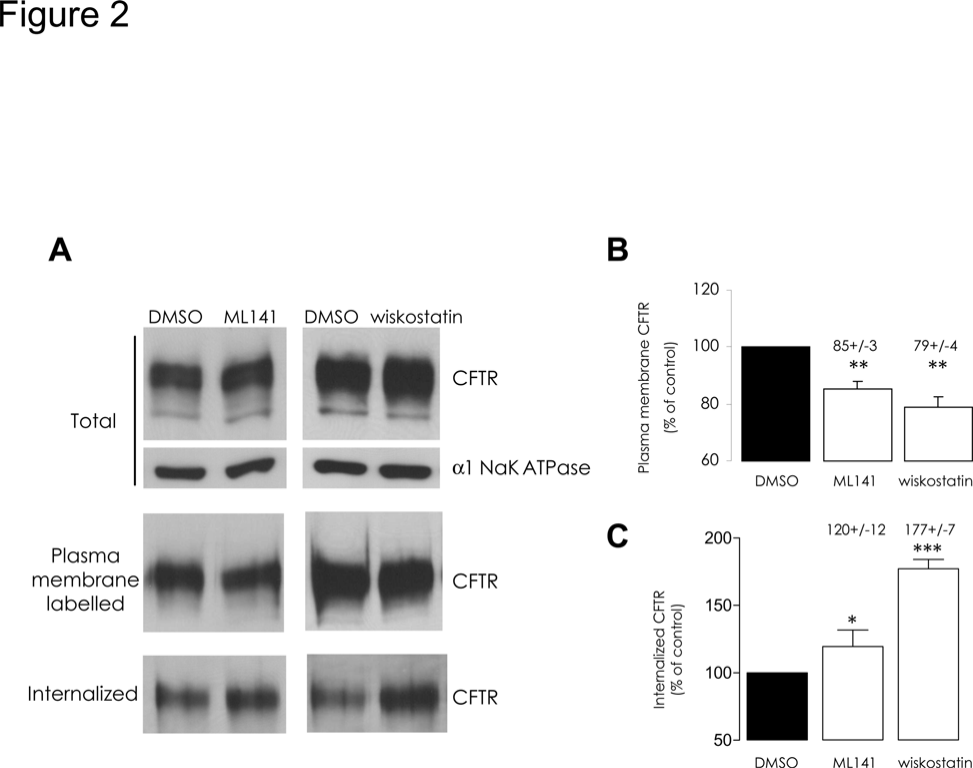
ML141 or wiskostatin treatments stimulate CFTR endocytosis. (A) Representative Western blots and (B and C) histograms summarizing the data are presented. First, cells were submitted to 10 μM ML141 for 30 min or 10 μM wiskostatin for 120 min. Treatment with 1% DMSO (v/v) was used as a negative control. Surface proteins were then biotinylated. Total CFTR protein amount was assessed by immunoblot in denatured samples obtained from 20 μg of clarified lysates (α1 Na^+^/K^+^ ATPase was used as a normalization control). Biotinylated proteins were purified from 100 μg of clarified lysates and the amount of labelled CFTR was assessed in the resulting samples. (B) The amount of plasma membrane CFTR, expressed as the percentage of DMSO treatment condition, decreased following both pharmacological treatments. Alternatively, the biotinylated PM proteins were allowed to enter the inner cell compartment through 5 min incubation of cell cultures at 37°C. Surface-exposed biotin moieties were then stripped by MESNA reduction. Biotinylated (internalized) proteins were purified from 600 μg clarified lysates and analyzed by Western blot. The ratio of the densitometric quantification of bands to the relative initial PM-CFTR amounts was then calculated. (C) Relative CFTR internalization, expressed as the percentage of DMSO control condition, appeared to have increased following ML141 or wiskostatin treatments. Data represent means ± SEM of 3 independent experiments, each performed in duplicate. ***: p<0.001, **: p<0.01, *: p<0.05.

**Fig 3:**
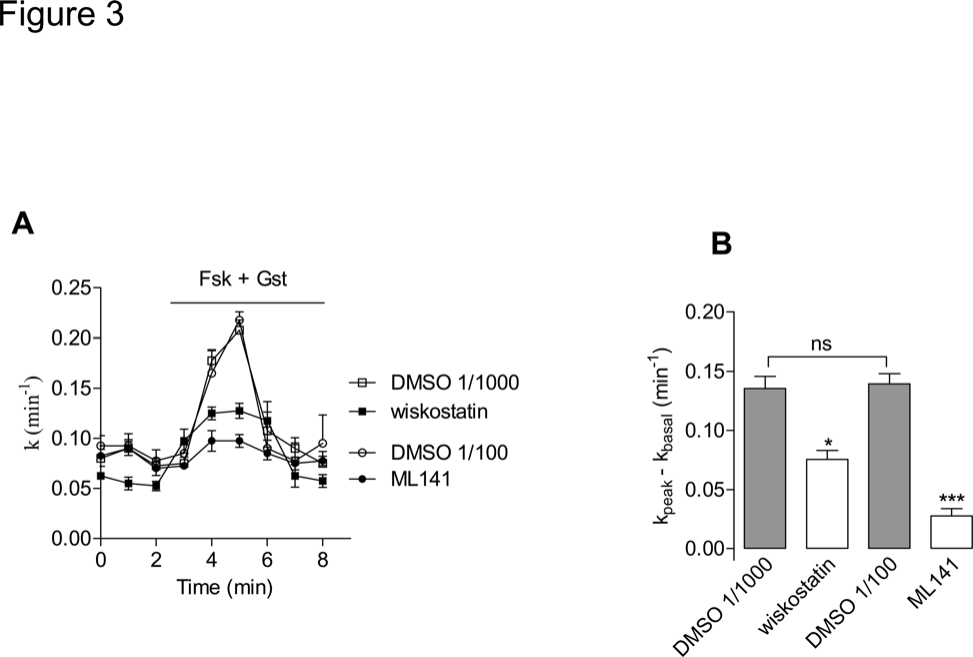
Pharmacological inhibitors of Cdc42 pathway impair CFTR channel activation. (A) Iodide efflux curves obtained in CFBE-wtCFTR cells treated with 10 μM wiskostatin for 120 min, 10 μM ML141 for 30 min or corresponding vehicle, prior to stimulation of CFTR activity by forskolin (Fsk, 10 μM) + genistein (Gst, 30 μM), n = 4 in each condition. (B) Histograms show the mean relative rates of CFTR activity. The result obtained with wiskostatin (resp. ML141) was compared with DMSO 1/1000 (resp. DMSO 1/100) (v/v) treatment. Means ± SEM are indicated. ***: p<0.001, *: p<0.05, ns: non significant.

Cells were exposed to either chemical, in the aforementioned conditions, and by means of a surface biotinylation assay we estimated the amounts of plasma membrane CFTR (PM-CFTR); both chemicals elicited a significant decrease of labelled PM-CFTR (Fig. 2A and 2B). To address the functional impact of this effect upon CFTR trafficking, we performed iodide efflux experiments after pharmacological inhibition of Cdc42 or of N-WASP in CFBE-wtCFTR cells; both ML141 and wiskostatin treatments led to a significant decrease of CFTR activation (Fig. 3) in accordance with our PM-CFTR quantification data.

### Pharmacological inhibitors of Cdc42 pathway increase dynamin 2-dependent endocytosis of CFTR

To investigate the dynamics of PM-CFTR, cells were treated with ML141 or wiskostatin compound at 37°C before performing surface protein biotinylation at 4°C; protein internalization was then allowed by returning the cells for 5 min at 37°C, in the presence of the drugs, in order to carry out endocytic assay. The amount of internalized CFTR was thereby slightly but significantly increased upon ML141 treatment and increased up to 77% with wiskostatin (Fig. 2A and 2C), in accordance with the previously observed lower PM-CFTR amount.

**Fig 4:**
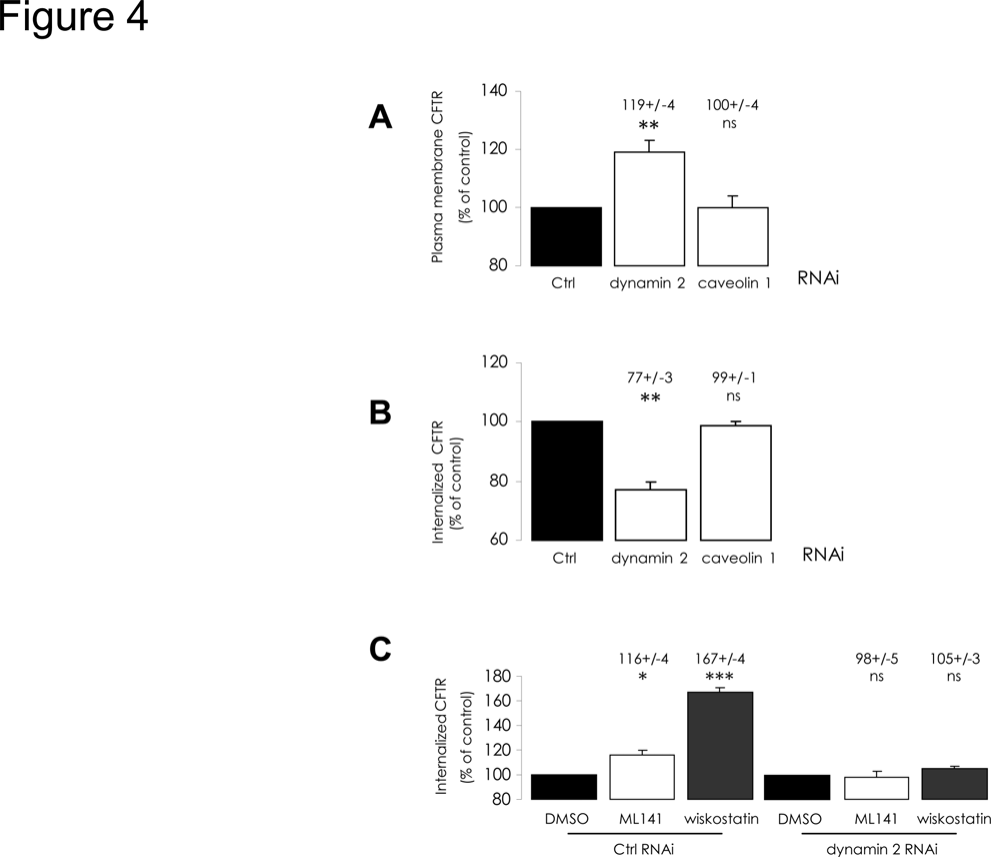
Alteration of dynamin 2-dependent mechanisms impairs the stimulation of CFTR endocytosis by ML141 or wiskostatin treatments. CFBE-wtCFTR cells were transfected with the corresponding siRNA to deplete dynamin 2 or caveolin 1 proteins 48 h before performing quantification of PM-CFTR or of internalized CFTR, as described in Fig. 2 legend. Histograms summarizing analyzed data are displayed. Results were compared with negative control siRNA transfection conditions. (A) Dynamin 2 depletion elicited an increase of PM-CFTR amount, (B) correlated to lower CFTR internalization, (A and B) whereas caveolin 1 depletion had no impact upon PM- and internalized CFTR amounts. Then, 48 h after transfection with indicated siRNA, cells were treated with 10 μM ML141 for 30 min or 10 μM wiskostatin for 120 min, before labelling and during the 5 min internalization period. Results were compared to vehicle treatment conditions. (C) When dynamin 2 is depleted, CFTR internalization increase upon pharmacological treatments is abolished. Data represent means ± SEM of 3 independent experiments, each performed in duplicate. ***: p<0.001, **: p<0.01, *: p<0.05, ns: non significant.

We then tried to determine which endocytosis pathway is involved during increased internalization. As dynamin is involved in clathrin- and caveolin-mediated endocytosis [24], we used siRNA-mediated depletion of dynamin 2 or caveolin 1 to interfere with the corresponding pathways. Knock-down efficiency was assessed by Western blot (S2 Fig.). As expected, while dynamin 2 depletion had no effect upon total CFTR amount (not shown), it elicited an increase in PM-CFTR abundance (Fig. 4A), correlated to a decrease in CFTR internalization (Fig. 4B). This finding highlights dynamin-dependent CFTR endocytosis in CFBE-wtCFTR cells. However, caveolin 1 depletion exhibited no impact upon either PM-CFTR amount (Fig. 4A), or CFTR internalization (Fig. 4B). This result suggests that caveolae do not mediate PM-CFTR uptake in these cells, and that dynamin-dependent endocytosis should occur mainly *via* clathrin-coated vesicles. When we treated dynamin 2-depleted cells with ML141 or wiskostatin, as described above, PM-CFTR amounts (not shown) and CFTR internalization were found to be unchanged compared to DMSO control (Fig. 4C). We concluded that treatments with these chemicals trigger increased clathrin-mediated internalization of CFTR.

### Pharmacological inhibitors of Cdc42 pathway decrease actin polymerization

**Fig 5:**
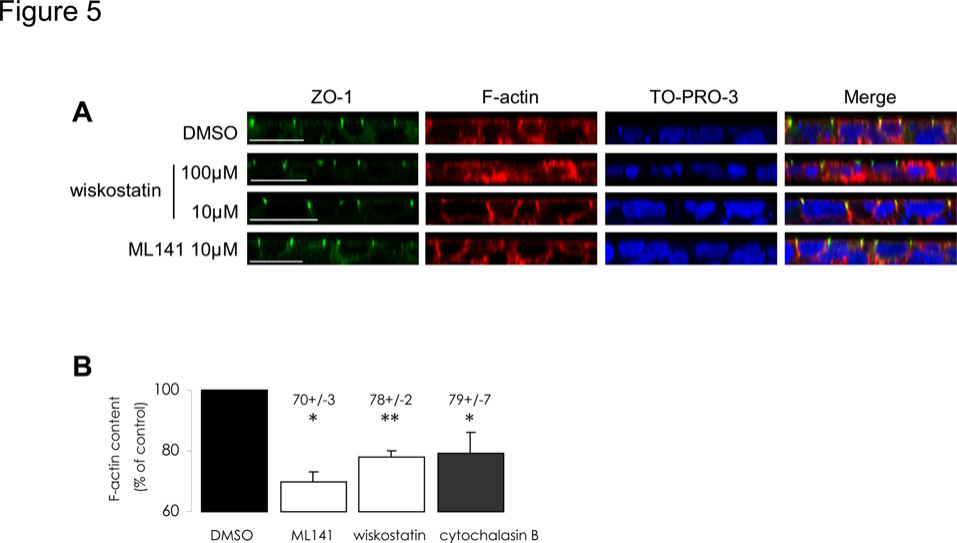
Pharmacological treatments decrease actin polymerization. (A) Cell shape, polarity and peripheral actin cytoskeleton pattern appear preserved upon 10 μM wiskostatin or 10 μM L141 treatments compared to vehicle treatment, whereas actin is scattered throughout the cytoplasm with 100 μM wiskostatin. Cells were grown on Transwell permeable support until a monolayer was established. After ML141 or wiskostatin treatments at the indicated concentrations, ZO-1 proteins were immunostained (green) and actin was tracked using phalloidin-TRITC (red). TO-PRO-3 was used as a cell nucleus marker (blue). Transversal section images were acquired with a confocal microscope. Scale bars represent 20 μm. (B) Pharmacological inhibitions of Cdc42 pathway reduce fibrillar (F-) actin content. Cells were incubated with 10 μM ML141 or 10 μM wiskostatin, and 10 μM cytochalasin B was used as an F-actin polymerization inhibition positive control treatment, whereas 1% DMSO (v/v) was used as negative control treatment. Actin-bound phalloidin-FITC was methanol-extracted and fluorescence measurements were performed. Results were normalized to protein amount and the relative F-actin contents are expressed as the percentage of DMSO control condition in histograms. Data represent means ± SEM of 3 independent experiments, each performed in triplicate. **: p<0.01, *: p<0.05.

As Cdc42 pathway is one of the regulation mechanisms involved in actin cytoskeleton remodelling, we checked the impact of previously applied treatments upon actin cytoskeleton integrity in CFBE-wtCFTR cells. First, we visualized the consequences of ML141 and wiskostatin treatments upon actin cytoskeleton organization by means of fluorescent confocal imagery, on a monolayer of polarized CFBE-wtCFTR cells grown on a permeable support (Fig. 5A). As depicted in transversal sections, ZO-1 immunostaining of tight junctions was not modified by the treatments. This further suggests that the polarity of the cells was not disrupted. In control condition (*i*.*e*. vehicle treatment), phalloidin staining highlighted cortical actin cytoskeleton above the apical and basolateral membranes. Upon 100 μM wiskostatin treatment, actin staining appeared blurry and scattered throughout the cell cytosol when compared to control. This severe disturbance of cytoskeleton organization is in accordance with the high level of cell mortality that we observed with the above-mentioned wiskostatin concentration. Conversely, in the presence of 10 μM wiskostatin or 10 μM ML141, the actin patterns within the cells appeared similar to the ones produced under control conditions.

We then quantified fibrillar (F-) actin amounts after pharmacological treatments: in both cases, we obtained a significant decrease in F-actin content (Fig. 5B). The values were comparable to the decrease elicited by cytochalasin B, a compound used to directly inhibit actin polymerization by blocking monomer addition. Even though the polarity and the actin filament scaffolding of the cells appeared unaltered at the imaging resolution we used, the polymerized actin amount was lowered upon treatments with these compounds. As discussed below, differences in actin polymerization status after pharmacological treatments may account for the destabilization of PM-CFTR, and hence for its increased internalization.

### Cdc42 depletion does not alter actin polymerization

**Fig 6:**
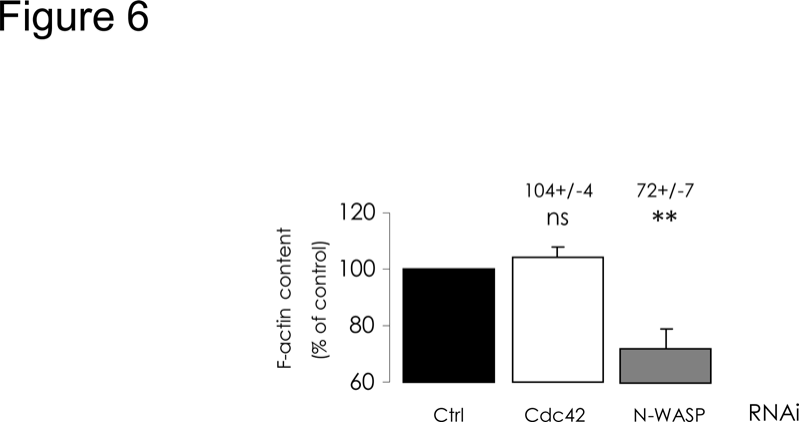
Cdc42 depletion does not alter fibrillar actin content. RNAi-mediated depletions of Cdc42 or N-WASP were performed for 48 h and F-actin content was quantified as described in Fig. 5 legend. As displayed in histograms, N-WASP depletion alone elicited a decrease of polymerized actin content, compared with negative control RNAi condition. Data represent means ± SEM of 3–8 independent experiments, each performed in triplicate. **: p<0.01, ns: non-significant.

To interfere with Cdc42 pathway in CFBE-wtCFTR cells, we applied a siRNA knock-down approach in order to deplete cells in the proteins that we had pharmacologically inhibited in the first part of our study. The efficiencies of these depletions were assessed by real-time RT-PCR and by Western blot (S3 Fig.). We first investigated the impact of these depletions upon polymerized actin content. As expected, N-WASP-depleted cells exhibited a significant decrease of F-actin amount (Fig. 6), and the consequences appear similar to those observed with wiskostatin inhibition of N-WASP. By contrast, when Cdc42 was depleted, no significant difference in F-actin content was observed when compared with cells transfected with negative control siRNA (Fig. 6), whereas ML141 inhibition of Cdc42 led to a decrease in polymerized actin content. Whatever may account for this difference, these findings suggest that alterations of CFTR trafficking resulting from Cdc42 depletion would not be triggered by actin scaffold-dependent mechanisms.

### Cdc42 depletion increases CFTR activity and total CFTR amount

**Fig 7:**
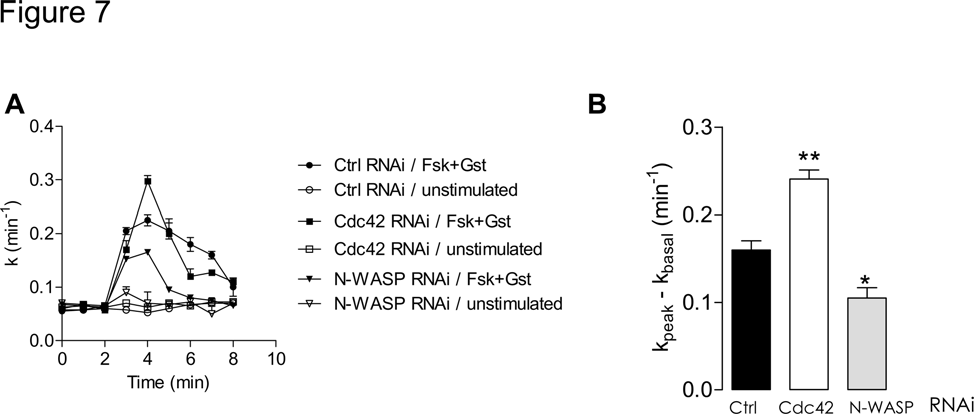
Cdc42 depletion increases CFTR channel activation. (A) In non-stimulated CFBE-wtCFTR cells (basal) or upon stimulation of CFTR activity by forskolin (Fsk, 10 μM) + genistein (Gst, 30 μM), iodide efflux curves were obtained after 48 h of siRNA-mediated depletions (negative control, Cdc42 or N-WASP), n = 4 in each condition. (B) Histograms show the mean relative rates of CFTR activity. Results obtained after Cdc42 and N-WASP depletions were compared to control RNAi condition. Means ± SEM are indicated. **: p<0.01, *: p<0.05.

We first investigated the impact of Cdc42 or N-WASP depletions upon CFTR channel function. We performed iodide efflux experiments 48 h after siRNA transfections: in N-WASP-depleted cells, CFTR activity decreased, whereas in Cdc42-depleted cells it increased (Fig. 7). In the first case, there are pronounced similarities to the consequences of wiskostatin treatment. As for Cdc42 depletion impact, it contrasts with the previously observed ML141inhibition of CFTR channel activation.

**Fig 8:**
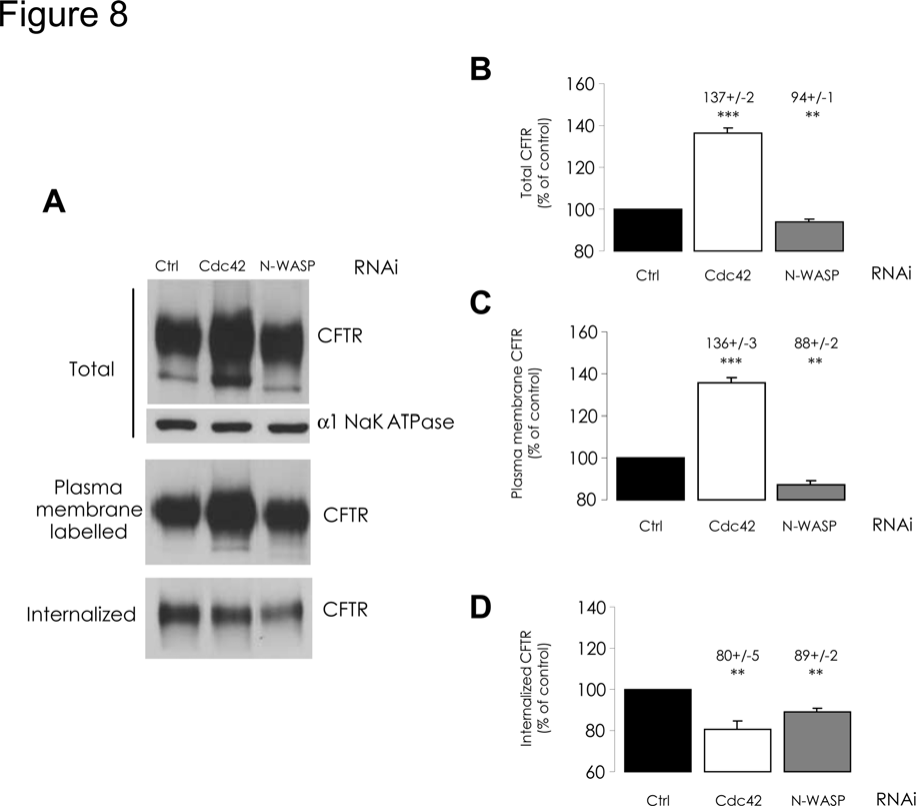
Impact of Cdc42 or N-WASP depletions upon CFTR amount and internalization. RNAi-mediated depletions of Cdc42 or N-WASP were performed for 48 h. We then used biotinylation experiments to evaluate CFTR amount at the cell surface and CFTR internalization, as described in Fig. 2 legend. (A) Representative Western blots and (B-D) histograms summarizing the data are presented. Following Cdc42 depletion, (B) total CFTR amount in whole cell lysate, (C) as well as CFTR amount at the plasma membrane, had increased compared to negative control RNAi conditions. (D) Internalized CFTR amount had decreased following Cdc42 depletion. Data represent means ± SEM of 3 independent experiments, each performed in duplicate. ***: p<0.001, **: p<0.01.

We then confronted these functional data with the quantification of CFTR protein amount: N-WASP depletion resulted in only a small decrease, while Cdc42 indeed elicited a 37% increase of total CFTR amount (Fig. 8A and 8B). Nevertheless, when protein synthesis was inhibited by cycloheximide for the last 24 h of the Cdc42 depletion, we observed no difference of total CFTR amount, compared to control (S4 Fig.). This suggests that Cdc42 may be involved in the stability of newly synthesized CFTR protein in early steps of its processing. We checked by real-time RT-PCR to make sure that neither of these depletions had an impact upon *CFTR* mRNA level (not shown), so the consequences may lie in post-transcriptional events such as translation, folding control, maturation or degradation of the protein. However, Western blots exhibited no apparent alteration of maturation from band B to band C, a process that terminates within the Golgi apparatus and appears unaffected.

Subsequently, by surface biotinylation assay we estimated the amounts of PM-CFTR in each condition. We found changes similar to those observed for total CFTR amount (Fig. 8A and 8C), the ratio of PM-CFTR to total CFTR remaining unaffected by Cdc42 or N-WASP depletion. This might suggest that steady-state PM-CFTR amount primarily reflects whole CFTR amount, whatever be the processing or trafficking step affected by Cdc42 pathway impairment. When Cdc42 was depleted, a small amount of the core-glycosylated form of CFTR (less than 5% of total CFTR) was detected in PM. Since we had previously checked that we did not label intracellular proteins (ezrin and β-actin) with our biotinylation protocol (not shown), this may be due to overexpression of CFTR.

### Cdc42 depletion does not alter CFTR maturation but decreases its degradation

**Fig 9:**
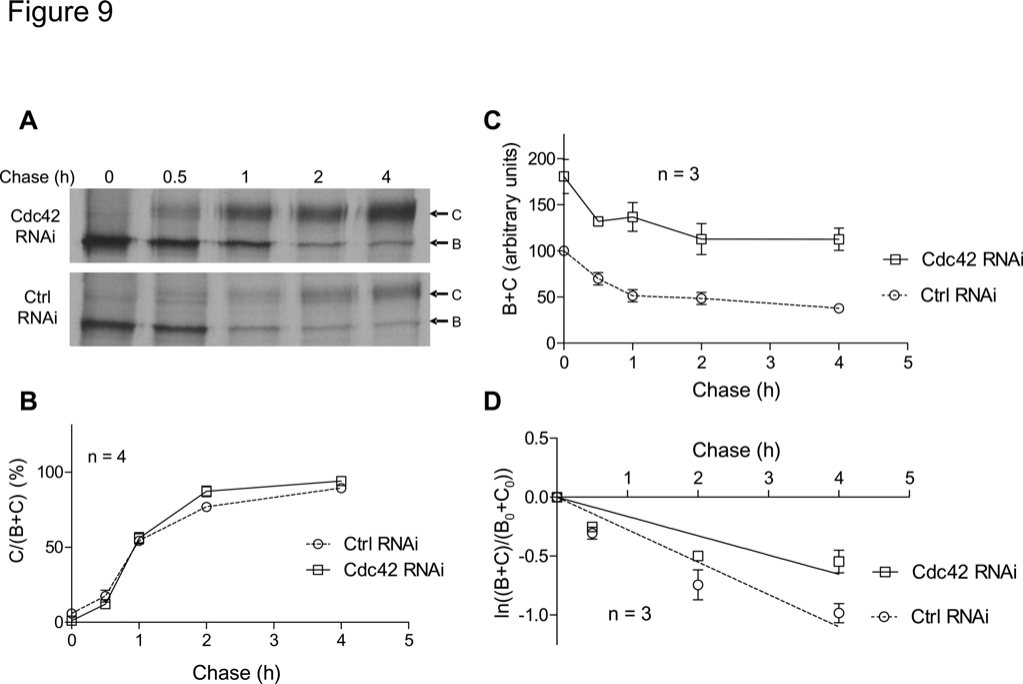
Analysis of CFTR maturation and turnover by metabolic labelling. CFBE-wtCFTR cells were transfected with negative control or Cdc42 siRNA and cultured 48 h prior to pulse-chase experiments. Cells were pulse-labelled for 15 min with 100 μCi/mL of [^35^S]methionine and [^35^S]cysteine mix and then chased for 0, 0.5, 1, 2, and 4 h. CFTR was then immunoprecipited and subjected to SDS-PAGE. Bands corresponding to core-glycosylated (band B) and fully-glycosylated (band C) CFTR were quantified by densitometry for analysis. (A) Representative gels are shown. (B) Maturation of CFTR is evaluated as the ratio of band C detected at a given time relative to total CFTR (bands B+C). (C) CFTR turnover is displayed as the relative total CFTR amount (bands B+C) along the chase. Total CFTR amount is assigned a value of 100 in arbitrary units at the beginning of the chase (0 h), when the cells are transfected with negative control siRNA. (D) The rate of CFTR disappearance is estimated as the natural logarithm of the amount of CFTR (bands B+C) at a given time of chase relative to its amount at the beginning of the experiment (B_0_+C_0_). Displayed lines are the linear regressions to the data. Symbol and error bars are means ± SEM of the values at each point. The numbers of independent experiments used to build the graphs are indicated on the figures.

To investigate the consequences of Cdc42 depletion upon CFTR processing and turnover, we performed pulse-chase experiments followed by CFTR immunoprecipitation (Fig. 9A). The time-course of CFTR glycosylation was estimated as the percentage of total CFTR that is fully-glycosylated (band C) along the chase (Fig. 9B). When comparing the curves corresponding to data obtained with negative control or Cdc42 RNAi, similar patterns were observed. The maturation statuses of CFTR at the end of the chase were found to be non-significantly different: C/(B+C) = 94.2±2.1% with Cdc42 depletion versus 89.4±1.5% in control conditions. In fact, Cdc42 did not appear to be involved in CFTR glycosylation steps occurring along the Golgi apparatus and should not be implicated in CFTR trafficking throughout this compartment.

We then used total CFTR amounts (bands B+C) along the chase to estimate CFTR turnover (Fig. 9C). When comparing the data obtained following negative control or Cdc42 RNAi, it was confirmed that total CFTR protein amount was increased by Cdc42 depletion. Moreover, in control conditions we estimated that 62.3% of CFTR was degraded during 4 h of chase, whereas when Cdc42 was depleted, the reduction was diminished to 38% of the initial CFTR amount. We next examined the rate of CFTR turnover as explained in legend to Fig. 9. According to the linear regression displayed in Fig. 9D, the slope of the line was-0.275±0.024 in control condition, increasing to-0.164±0.020 when Cdc42 was depleted. This suggests that siRNA-mediated Cdc42 impairment leads to a slowdown of CFTR turnover in the first hours of its processing, and hence that Cdc42 pathway is likely to promote the degradation of some newly synthesized CFTR.

### Impact of Cdc42 depletion upon CFTR dynamics at plasma membrane

As we did before with total CFTR, we estimated the variations of PM-CFTR amount when protein synthesis was inhibited for the last 24 h of siRNA treatments. Under Cdc42 depletion, the apparent PM-targeting efficiency of CFTR was decreased (S5 Fig.). As this cycloheximide treatment impaired the augmentation of total CFTR amount following Cdc42 depletion (S4 Fig.), this could be due to a decrease of the CFTR supply to the secretion process. This confirms that under Cdc42 depletion, CFTR proteins which are stabilized in the early steps of their processing are targeted to PM.

**Fig 10:**
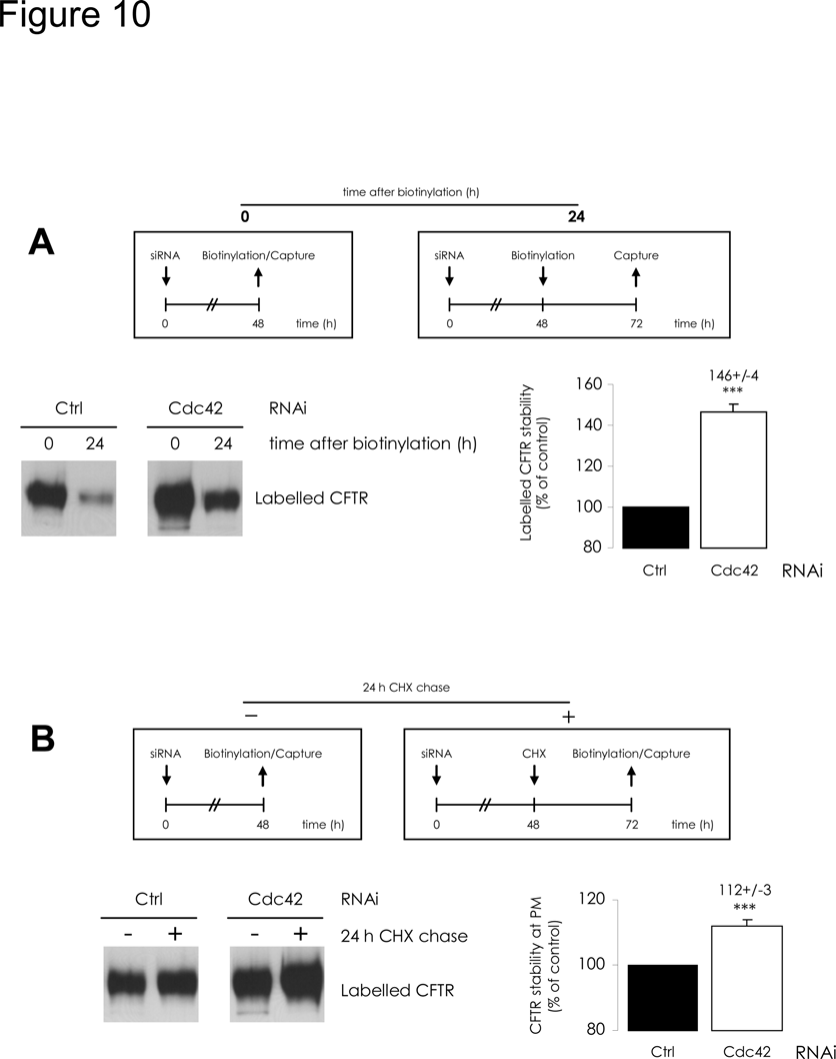
Cdc42 depletion increases CFTR stability at plasma membrane. Two approaches were used to assess CFTR turnover downstream of its targeting to plasma membrane. (A) Cdc42 depletion increases stability of the PM-targeted CFTR. The upper diagrams summarize the procedures followed. 48 h after cell transfection by the mentioned siRNA, surface proteins were labelled. Streptavidin capture occurred either subsequently (0), or after a 24 h incubation of cell cultures at 37°C (24). Labelled CFTR protein amounts were assessed in the resulting samples. After densitometric quantification of Western blot images (representative examples in the bottom left panel), (24) to (0) ratios were calculated. The Ctrl RNAi value was used to define 100% of labelled CFTR stability. In the bottom right panel, the histogram shows that the relative PM-CFTR stabilities increase when Cdc42 is depleted. (B) Cdc42 depletion increases remaining PM-CFTR after cycloheximide chase. The upper diagrams summarize the procedures followed. 48 h after cell transfection by siRNA, the PM proteins were labelled and purified (-). Alternatively, the cell cultures were submitted to additional 24 h incubation with 100 μg/mL cycloheximide (+), before biotinylation and capture were performed. The labelled CFTR protein amounts were assessed by densitometric quantification of Western blot bands (representative images in the bottom left panel). In both RNAi conditions, labelled CFTR amounts extracted from the same amount of whole cell lysates appeared higher after 24h CHX chase: this could be explained by stability differences between the various cellular proteins. To overcome this bias, we compared (+) to (-) ratios and the Ctrl RNAi value was used to define 100% of CFTR apparent PM stability. In the bottom right panel, histogram shows that CFTR stability at PM appears higher when Cdc42 is depleted. Data represent means ± SEM of 3 independent experiments, each performed in duplicate. ***: p<0.001.

Independently of Cdc42 pathway implication in the early steps of CFTR processing, we tried to determine whether it might be involved in distal trafficking steps of CFTR *i*.*e*. downstream of its exit from the Golgi apparatus. To evaluate variations in CFTR stability upon depletion of Cdc42, we used two approaches. On the one hand, we estimated the degradation rate of the CFTR initially expressed at the plasma membrane: PM-CFTR was biotinylated, and the labelled CFTR amount was quantified at the beginning of the experiment and after 24 h of further cell incubation in culture medium at 37°C. The percentage of remaining labelled CFTR after the 24 h chase is an indicator of the stability of initial PM-CFTR; it increased when Cdc42 was depleted compared to control (Fig. 10A). On the other hand, we determined the variations of PM-CFTR amount between a condition in which we labelled surface proteins and extracted them 48 h after siRNA transfection, and a condition in which labelling and extraction were performed after 24 h of supplementary CHX chase. These CHX treatment conditions do not preclude the effects of Cdc42 depletion upon early steps of CFTR processing. Consequently, the difference should result only from an equilibrium between endocytosis, recycling and degradation. Cdc42 depletion then elicited an increase of remaining PM-CFTR, in comparison to control (Fig. 10B). Both results are consistent with the hypothesis of improved CFTR stability in PM when the Cdc42 pathway is impaired.

**Fig 11:**
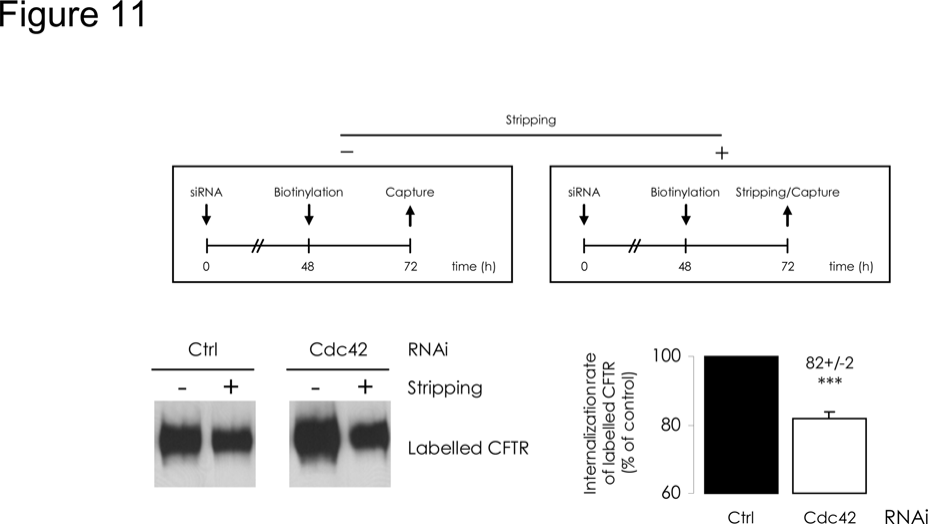
Cdc42 depletion reduces the long-term CFTR internalized fraction. The upper diagrams summarize the procedures followed. 48 h after cell transfection by siRNA, PM proteins were labelled and the cultures were submitted to additional 24 h incubation at 37°C. Purification occurred either directly (-), from 200 μg clarified lysates, or alternatively, after a MESNA-mediated stripping step (+), from 600 μg clarified lysates. After densitometric quantification of Western blot images (representative example in the bottom left panel), (+) to 3×(-) ratios were calculated. The Ctrl RNAi value was used to define 100% of long-term internalized fraction. In the bottom right panel, histograms express the relative CFTR internal fractions normalized to the Ctrl RNAi condition. Data represent means ± SEM of 3 independent experiments, each performed in duplicate. ***: p<0.001.

In addition, we evaluated CFTR internalization with an endocytic assay: surface proteins were labelled 48h after siRNA transfection and 5 min incubation at 37°C triggered internalization. Several studies [25,26] have shown that reactivating intracellular trafficking for 5 min allows for CFTR endocytosis measurement before recycling or degradation may interfere. After stripping of the labelled protein remaining at the cell surface, intracellular CFTR was streptavidin-bound and quantified. Internalization rate was then reduced by 20% upon Cdc42 depletions (Fig. 8D). We then used another protocol to assess longer term effect upon internalized CFTR amount; labelling was performed 48 h after siRNA transfection and cells were further cultured for 24 h; either whole labelled CFTR (at PM and intracellular) or labelled CFTR remaining inside cellular compartments (protected from stripping) were then captured and quantified. The ratio of intracellular to total biotinylated CFTR was calculated. The relative fraction of internal labelled CFTR appeared to have been lowered upon Cdc42 depletion (Fig. 11). This suggests that the decrease of CFTR endocytosis was sustained during 24 h or was perhaps balanced by increased recycling.

## Discussion

The role of the small GTPase Cdc42 in endocytosis and recycling phenomena has been highlighted in numerous cell models [16,17]. Whether Cdc42 attends dynamin-dependent endocytosis (mediated by clathrin-coated vesicles or caveolae) or poorly characterized dynamin-independent pathways remains debatable and is likely to depend on the cell context. Amongst the Cdc42 downstream effectors of which the activation is required in these internalization processes, N-WASP and Toca-1 have been extensively studied [27]. In CFTR-transfected BHK cells, pharmacological inhibition of N-WASP has been shown to result in a decrease of CFTR cell surface expression, *via* accelerated endocytosis and seemingly impaired recycling [18]. In our work, we investigated the involvement of the Cdc42 signalling pathway upstream from the N-WASP effector in CFTR steady-state and in its trafficking, to and from plasma membrane (PM), in a human bronchiolar epithelial cell line overexpressing wild-type CFTR (CFBE-wtCFTR) [19].

Two distinct approaches were chosen to investigate the consequences upon CFTR homeostasis of Cdc42 pathway impairments: (i) we exposed cells to the pharmacological inhibitor ML141, a new compound selectively inhibiting Cdc42 [20], or to wiskostatin; (ii) we performed depletions by RNA-interference of either Cdc42 itself or else its effector N-WASP. Consequences upon CFTR chloride channel activity were studied by iodide efflux assay. Through various biotinylation-based assays, we estimated the impact of these treatments upon CFTR total and cell surface amounts, plasma membrane stability and internalization. We thereby showed that Cdc42 pathway is involved in CFTR turnover as well as its PM expression and dynamics.

Apparent discrepancies between results appeared (i) when we compared data obtained by knocking down a protein or by its pharmacological inhibition, and (ii) when we depleted cells of Cdc42 or of its effector N-WASP. However, assuming that Cdc42 activation, whether involved in internalization of endocytic vesicles or in other mechanisms, results in actin cytoskeleton remodelling, we observed that our results depend on actin polymerization state within the cells: Cdc42 depletion did not modify F-actin content in the cells, whereas N-WASP depletion and pharmacological treatments did. Moreover, CFTR trafficking appeared differently modified in cells with no apparent modification of actin cytoskeleton organization compared with cells in which actin polymerization was disturbed.

Interplay between CFTR and actin cytoskeleton has been extensively studied. A consensus has emerged, according to which proper actin organization is required for efficient CFTR channel activation, even though conflicting results have been published concerning the effect of actin-depolymerizing chemicals [28–30]. This may depend on a balance between the cortical cytoskeleton integrity necessary to the stability of CFTR at plasma membrane and the requirement of actin microfilaments to internalize unstable CFTR protein by endocytosis vesicles. The anchoring of PM-CFTR to actin microfilaments was shown to occur *via* the binding of its carboxy-terminal cytoplasmic domain to several proteins harbouring PDZ (PSD-95/Dlg/ZO-1) domain. These interactions were shown to be involved in CFTR chloride channel activation [31,32]. Experiments based on C-terminus truncation of CFTR have fed controversies about the requirement of PDZ interactions in CFTR endocytosis and recycling [33–35]. However, overexpression of one of these proteins, the Na^+^/H^+^ exchanger regulatory factor isoform 1, was shown to increase the stability at PM of the rescued F508del-CFTR mutant [36].

In our experiments, the pharmacological inhibitions of Cdc42 or of N-WASP seem to elicit a loosening of cortical actin network without globally disrupting actin cytoskeleton organization. Moreover, we have observed a decrease of PM-CFTR amount that was correlated to an increase of CFTR internalization, *via* dynamin- (and presumably clathrin-) dependent endocytosis. As a result, when cells are treated with ML141 or wiskostatin, CFTR is likely to be less stably anchored to cytoskeleton and to become more prone to undergo endocytosis. As PM-CFTR amounts were finally reduced, internalization would not be counterbalanced by recycling. These results are consistent with the work describing the effect of wiskostatin treatment in CFTR-transfected BHK cells [18].

Another issue in use of chemical inhibitors is their specificity. Concerning wiskostatin, additional effects distinct from N-WASP inhibition have been observed [37,38]; it has been shown to alter global cell functions by decreasing intracellular ATP level. However, this effect was observed primarily with concentrations higher than 10 μM, which we used in our study. As for ML141, its inhibitory selectivity has been checked in a selection of the most widely studied small GTPases but to our knowledge, alternative effects on other cellular process have not been investigated. Wiskostatin and ML141 may have uncharacterized impacts upon actin cytoskeleton or CFTR trafficking, unrelated to Cdc42 pathway impairment. This problem should be resolved by siRNA-mediated depletion of the involved proteins.

The fact that depleting Cdc42 or N-WASP, which are two actors of the same signalling pathway, elicits dissimilar consequences upon F-actin content remains to be explained. In the case of N-WASP knock-down, disruption of a pathway leading to actin branching is consistent with the results we obtained. Nevertheless, after 48 h depletion of Cdc42, N-WASP may still be activated by other cues such as phosphoinositide lipids, SH3 domain-containing proteins or by phosphorylation [39]. Moreover, the cells might then use compensation mechanisms to reorganize their cytoskeleton. However, when we chemically inhibited Cdc42 or N-WASP, a decrease of F-actin content was obtained. As drug treatment durations were much shorter (maximum 2 h) than the 48 h siRNA-mediated depletion times, no putative cytoskeleton polymerization recovery was allowed to occur when we performed the subsequent functional or biochemical assays. Moreover, we observed by fluorescent imaging that cortical actin localization was identical to control condition at the compound concentration we used. The cytoskeleton should remain organized, even if it is less tightly polymerized.

When we performed siRNA-mediated depletion of N-WASP, the results were similar to those observed with wiskostatin treatment: F-actin content was reduced and inhibition of CFTR channel activation was consistent with a decrease of PM-CFTR amount. In this case, inhibitions of N-WASP by pharmacological treatment or by protein depletion likely trigger similar actin-mediated destabilization of CFTR at PM. In contrast, siRNA-mediated depletion of Cdc42 has different consequences than pharmacological inhibition of Cdc42 with ML141 upon CFTR. Moreover, we can assume that the impact upon CFTR that we subsequently observed was independent of actin polymerization status and of CFTR stabilization at PM *via* its anchoring to cortical cytoskeleton. Cdc42 depletion elicited a significant increase of total CFTR amount: this was highlighted by the larger amount of CFTR protein synthesized during 15 min of metabolic labelling. By Western blot, we noted sustained augmentation of steady-state protein amount. As we found no significant change in *CFTR* mRNA level using real-time RT-PCR, it can be inferred that Cdc42 is not involved in the regulation of *CFTR* transcription. One cannot exclude the notion that the rate of CFTR translation may be involved, as it has been noted that Cdc42 can activate the 70 kDa S6 kinases, which are components of the 40S ribosomal subunit, and are involved in protein translation modulation [40]. However, pulse-chase data show that the initial rate of CFTR degradation is slowed down upon Cdc42 depletion. The fact that at the beginning of the chase, core-glycosylated CFTR (band B) amount increases by 80% suggests that Cdc42 depletion leads to escape from endoplasmic reticulum- (ER-) associated protein degradation (ERAD) during the first steps of maturation and folding control. The absence in these experiments of observed consequences upon Na^+^/K^+^ ATPase α1 subunit supports the idea that the mechanism that is implicated affects mostly the ER quality control process of proteins of which the folding is poorly efficient (as CFTR). Therefore, Cdc42 pathway seems necessary to ERAD targeting, in a manner that remains to be investigated.

When we examined the time-course of CFTR maturation by pulse-chase experiments, we observed no alteration in the conversion of core-glycosylated into a fully-glycosylated form of CFTR (band B to band C). As soon as CFTR has been exported out of the ER, it appears that Cdc42 pathway does not attend CFTR trafficking through the Golgi apparatus.

We subsequently evaluated whether Cdc42 pathway is implicated in distal CFTR trafficking events, *i*.*e*. after the protein has escaped ERAD and has undergone full glycosylation in the Golgi. We observed an improvement of CFTR stability at the cell surface upon Cdc42 depletion. This can be explained by a decrease of CFTR internalization, estimated for 5 min after surface protein labelling. This short time lapse allows for quantification of endocytosis before recycling and lysosomal degradation can occur. When we quantified CFTR initially labelled in PM and remaining intracellular 24 h later, we observed that Cdc42 depletion continued to induce a decrease, similar to the one obtained after 5 min internalization, thereby suggesting sustained diminishing of endocytosis. Moreover, since we observed no accumulation over time of internalized CFTR within the cells, the protein should be sorted toward recycling rather than toward lysosomal degradation. Taken together, these data suggest that the Cdc42 pathway promotes the lysosomal degradation of internalized PM-CFTR.

To date, only one Rho family small GTPase, TC10, has been reported to be involved in CFTR trafficking, by up-regulating total and PM-CFTR amounts [41]. Following the full maturation of CFTR in the Golgi apparatus, TC10 disrupts interactions between CFTR and CAL, a PDZ domain protein mediating CFTR targeting to lysosomal degradation. In this study, the authors transfected COS-7 cells with vectors encoding constitutively active and dominant-negative forms of small GTPases: they concluded that only TC10, but not Cdc42, attended CFTR regulation. We had nevertheless begun our work by expressing these mutant forms of Cdc42 protein in CFBE-wtCFTR cells, but the relevant experiments appeared inconclusive. Actually, the fact that use of wild type and mutant forms of small GTPases may in some contexts conceal their genuine involvement in several cellular processes has already been mentioned and discussed [42]. For instance, overexpressed proteins may sequestrate the factors that should regulate the activation/inactivation cycle of endogenous small GTPases. We consequently applied the RNAi-mediated depletion approach in our study.

Concerning Cdc42 pathway, we wish to suggest that it promotes CFTR degradation by a dual mechanism: first, it contributes to co- or post-translational degradation of CFTR *via* ERAD. To our knowledge, no connection between these steps of protein processing and Cdc42 has been studied in previously published work. However, our data do not imply that Cdc42 involvement should be direct. In fact, there is a large amount of redundancy in the interactions between small GTPases and (i) their effectors or (ii) their regulator proteins (guanine nucleotide exchange factors, GTPase activating proteins and GDP disassociation inhibitors). Depleting the cells in Cdc42 may unleash some of these proteins and allow them to interfere with the regulation of other small GTPases, which may be involved in the results that we observed. For instance, RhoD has been shown to attend intracellular membrane trafficking, contributing to Golgi homeostasis, which is apparently not involved in our study, but is indeed involved in ER to Golgi transport and endosome trafficking [43]. The second processing step of CFTR in which Cdc42 appears to be implicated is endocytosis and sorting to lysosomal degradation rather than recycling, in agreement with published data concerning Cdc42 involvement in vesicle trafficking [16,17]. Investigating the contribution of Cdc42 pathway to the discarding of misfolded CFTR proteins could lead to clues as how to increase PM expression of trafficking-defective mutant forms of CFTR.

## Supporting Information

S1 Fig Pharmacological inhibitions of Cdc42 pathway decrease the relative PM-CFTR amount. Cells were cultured in the presence of ML141 or wiskostatin at the indicated concentrations for the indicated times. Treatment with 1% DMSO (v/v) was used as a negative control. The relative PM-CFTR amounts, expressed as the percentage of control condition in the histograms, were obtained as in Fig. 2B. Data represent means ± SEM of 3 independent experiments each performed in duplicate. **: p<0.01, ns: non-significant.
(TIF)

S2 Fig RNAi-mediated dynamin 2 or caveolin 1 depletion efficiencies. Cells were transfected with the mentioned siRNA. 48 h later, ezrin, dynamin 2, caveolin 1 and β-actin protein amounts were assessed in denatured samples obtained from 20 μg of clarified lysates. For each protein, after densitometric quantification of Western blot images (representative example in the upper panel), ratio to β-actin was calculated. Relative protein amounts were expressed as % of Ctrl RNAi condition. The lower histograms express the resulting depletion efficiencies. Data represent means ± SEM of at least 3 independent experiments each performed in duplicate. ***: p<0.001.
(TIF)

S3 Fig RNAi-mediated Cdc42 and N-WASP depletion efficiencies. Cells were transfected with the mentioned siRNA and incubated for 48 h. (A) Relative mRNA amount were estimated by real-time RT-PCR. (B) Protein amounts were assessed in denatured samples obtained from 20 μg of clarified lysates. For each protein, after densitometric quantification of Western blot images (representative examples are displayed), ratio to β-actin was calculated. The relative protein amounts were expressed as the percentage of Ctrl RNAi condition. Histograms express the resulting depletion efficiencies. Data represent means ± SEM of at least 3 independent experiments each performed in duplicate. ***: p<0.001, **: p<0.01.
(TIF)

S4 Fig Increase of total CFTR following Cdc42 depletion is impaired by cycloheximide treatment. The upper diagram summarizes the procedures followed. Cells were transfected with the corresponding siRNA to deplete Cdc42 protein and incubated for 48 h. In addition, cells were exposed to 100 μg/mL cycloheximide for the last 24 h. The CFTR and α1 NaK ATPase protein amounts were then assessed in denatured samples obtained from 20 μg of clarified lysates. Representative Western blot image is shown. Densitometric quantification of bands was normalized to Ctrl RNAi value. Total CFTR relative amounts are expressed as % of control condition in the histogram. Data represent means ± SEM of at least 3 independent experiments, each performed in duplicate. ns: non-significant.
(TIF)

S5 Fig Cdc42 depletion decreases apparent PM-targeting efficiency of CFTR. The upper diagrams summarize the procedure followed. Cells were siRNA-transfected to deplete Cdc42 protein and cultured for 48 h. In addition, cells were exposed to 100 μg/mL cycloheximide (+), or 0.1% DMSO (v/v) for the control condition (-), for the last 24 h. Afterwards, PM proteins were biotinylated and purified from 100 μg clarified lysates. Labelled CFTR protein amounts were assessed in the resulting samples by densitometric quantification of Western blot images (representative examples in the bottom left panel). In control RNAi condition, labelled CFTR amounts extracted from the same amount of whole cell lysates appeared higher after 24h CHX treatment: stability differences between the various cellular proteins may account for this paradox. We estimated PM-targeting efficiency by calculating (+) to (-) ratios, the Ctrl RNAi value being used as 100% of apparent PM-targeting efficiency. In the bottom right panel, histogram expresses the relative CFTR cell surface targeting efficiency. Data represent means ± SEM of 3 independent experiments, each performed in duplicate. **: p<0.01.
(TIF)

S1 Table siRNA sequences. (DOC)
