# Supplementary figures and images for: Involvement of the Cdc42 Pathway in CFTR Post-Translational Turnover and in Its Plasma Membrane Stability in Airway Epithelial Cells
Source: PLoS One. 2015 Mar 13;10(3):e0118943. doi: 10.1371/journal.pone.0118943 (PMC4359135; doi:10.1371/journal.pone.0118943)

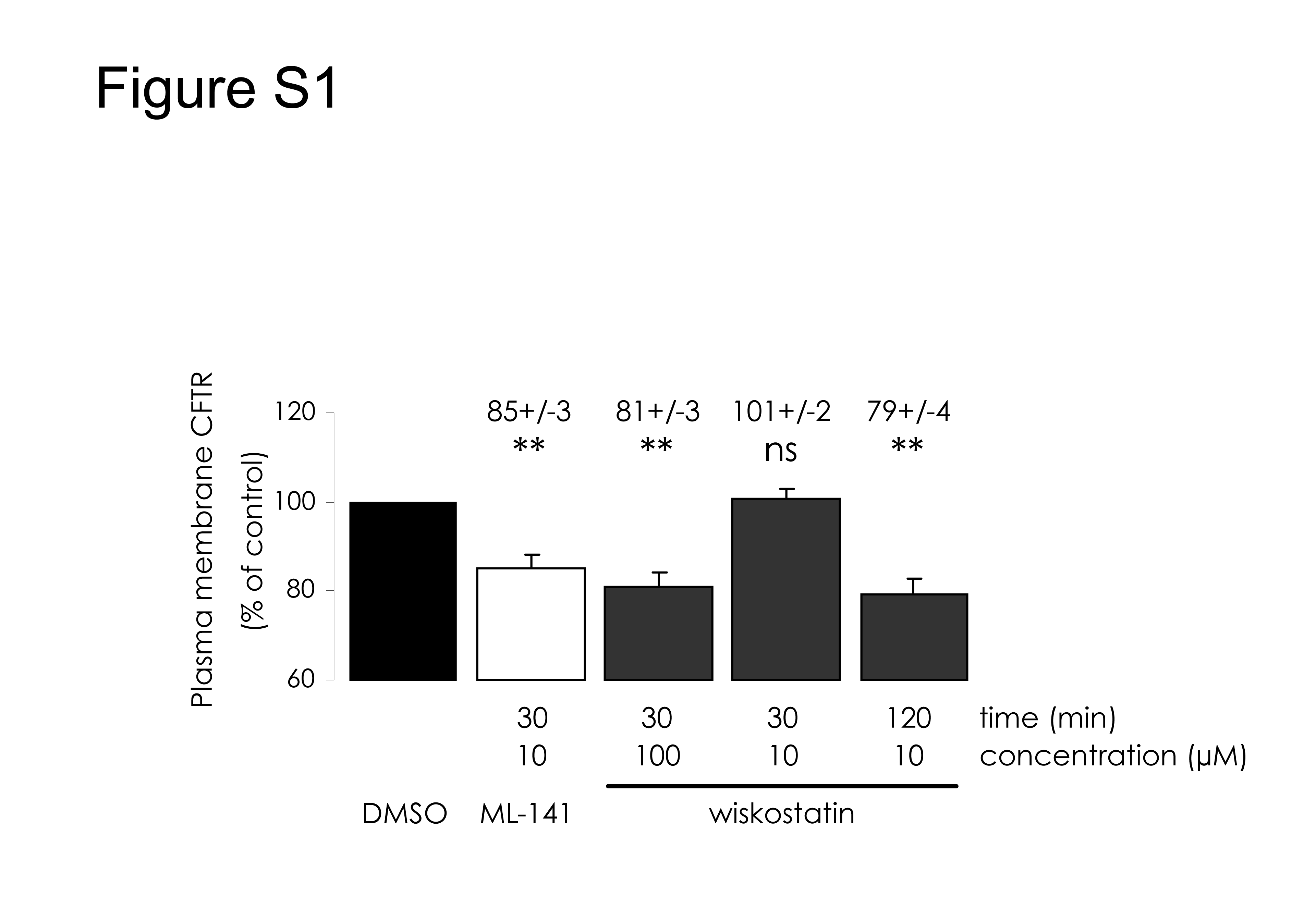

Supplement: S1 Fig — Cells were cultured in the presence of ML141 or wiskostatin at the indicated concentrations for the indicated times. Treatment with 1% DMSO (v/v) was used as a negative control. The relative PM-CFTR amounts, expressed as the percentage of control condition in the histograms, were obtained as in Fig. 2B. Data represent means ± SEM of 3 independent experiments each performed in duplicate. **: p<0.01, ns: non-significant. (TIF) [file pone.0118943.s001.tif]

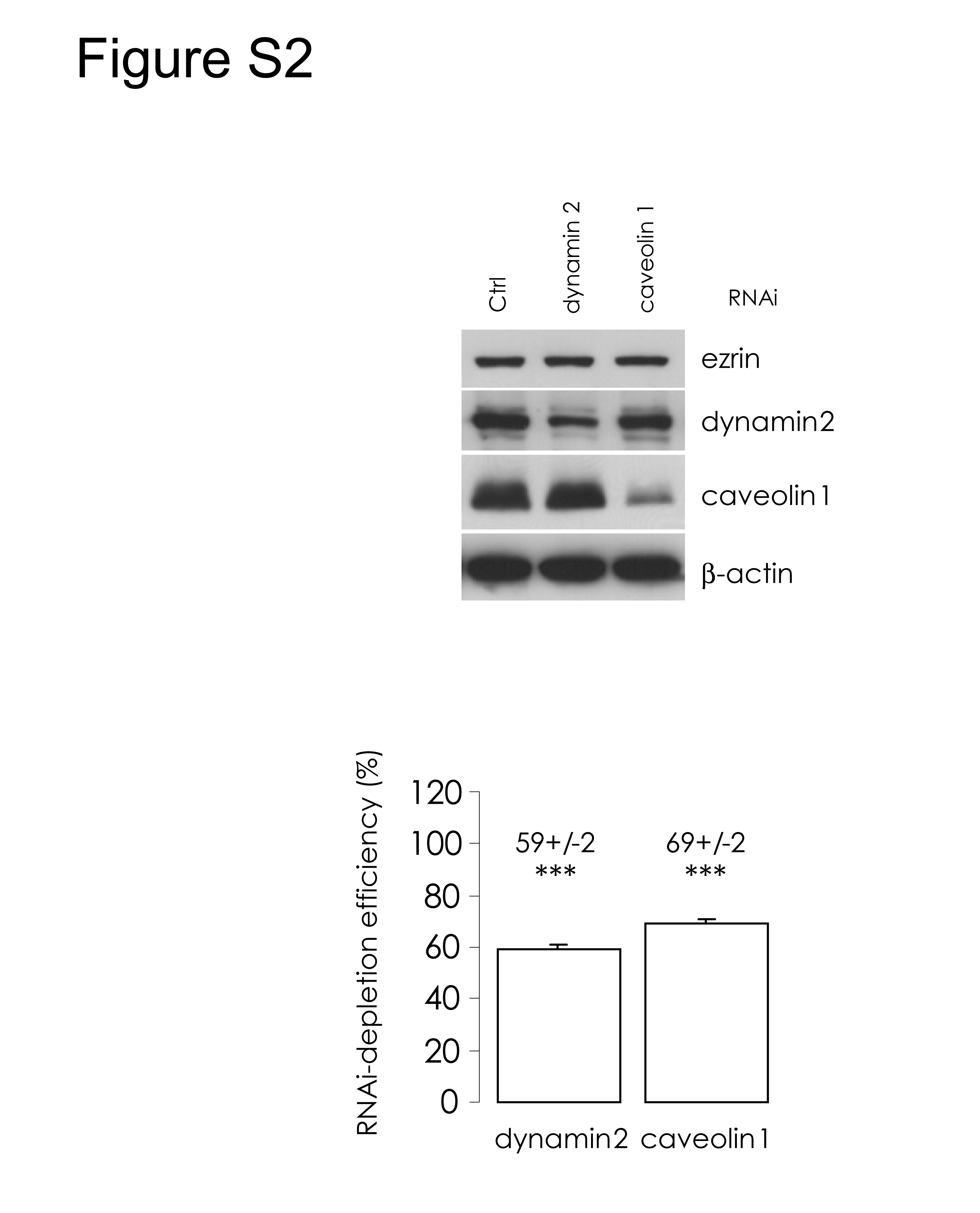

Supplement: S2 Fig — Cells were transfected with the mentioned siRNA. 48 h later, ezrin, dynamin 2, caveolin 1 and β-actin protein amounts were assessed in denatured samples obtained from 20 μg of clarified lysates. For each protein, after densitometric quantification of Western blot images (representative example in the upper panel), ratio to β-actin was calculated. Relative protein amounts were expressed as % of Ctrl RNAi condition. The lower histograms express the resulting depletion efficiencies. Data represent means ± SEM of at least 3 independent experiments each performed in duplicate. ***: p<0.001. (TIF) [file pone.0118943.s002.tif]

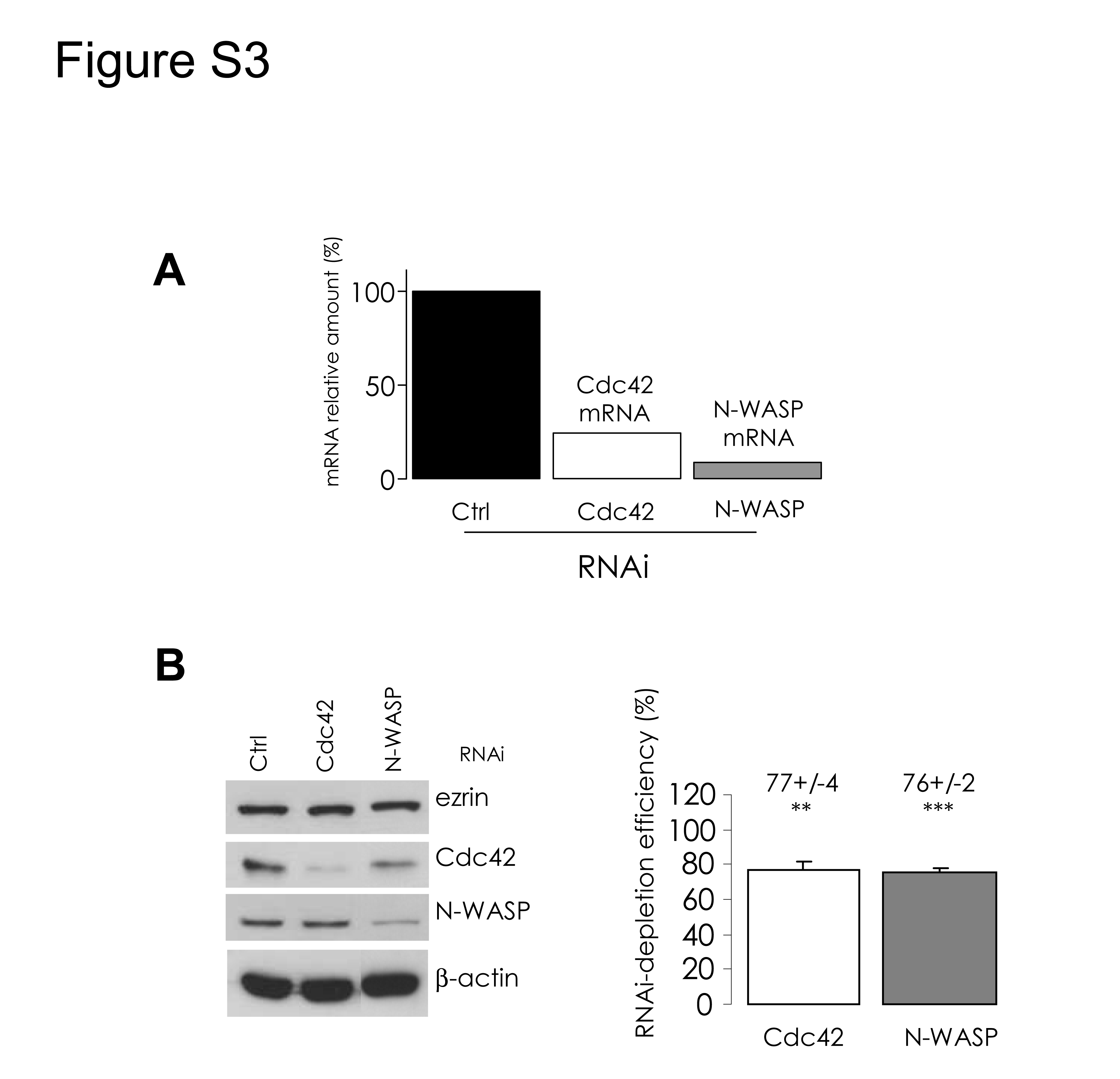

Supplement: S3 Fig — Cells were transfected with the mentioned siRNA and incubated for 48 h. (A) Relative mRNA amount were estimated by real-time RT-PCR. (B) Protein amounts were assessed in denatured samples obtained from 20 μg of clarified lysates. For each protein, after densitometric quantification of Western blot images (representative examples are displayed), ratio to β-actin was calculated. The relative protein amounts were expressed as the percentage of Ctrl RNAi condition. Histograms express the resulting depletion efficiencies. Data represent means ± SEM of at least 3 independent experiments each performed in duplicate. ***: p<0.001, **: p<0.01. (TIF) [file pone.0118943.s003.tif]

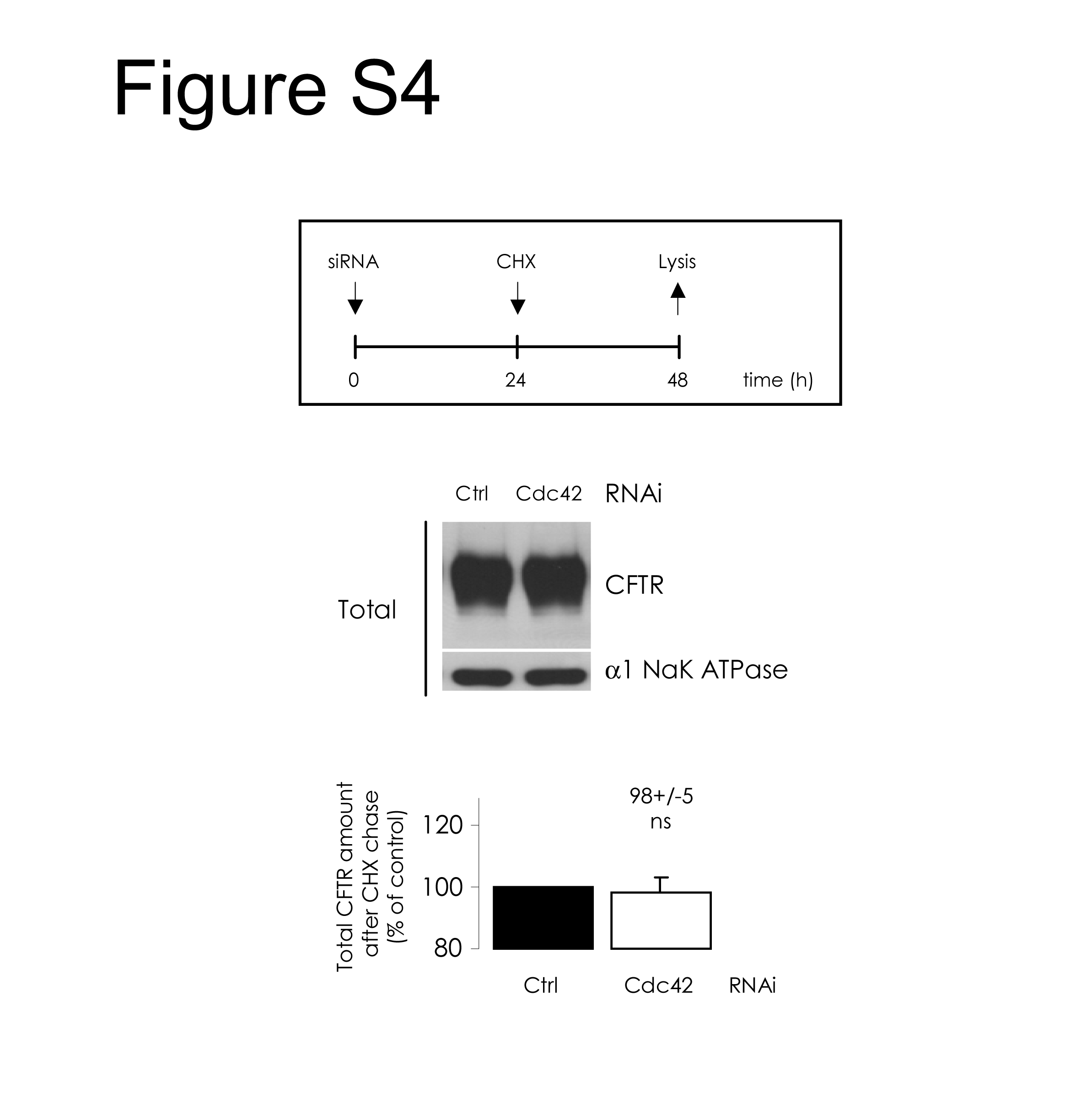

Supplement: S4 Fig — The upper diagram summarizes the procedures followed. Cells were transfected with the corresponding siRNA to deplete Cdc42 protein and incubated for 48 h. In addition, cells were exposed to 100 μg/mL cycloheximide for the last 24 h. The CFTR and α1 NaK ATPase protein amounts were then assessed in denatured samples obtained from 20 μg of clarified lysates. Representative Western blot image is shown. Densitometric quantification of bands was normalized to Ctrl RNAi value. Total CFTR relative amounts are expressed as % of control condition in the histogram. Data represent means ± SEM of at least 3 independent experiments, each performed in duplicate. ns: non-significant. (TIF) [file pone.0118943.s004.tif]

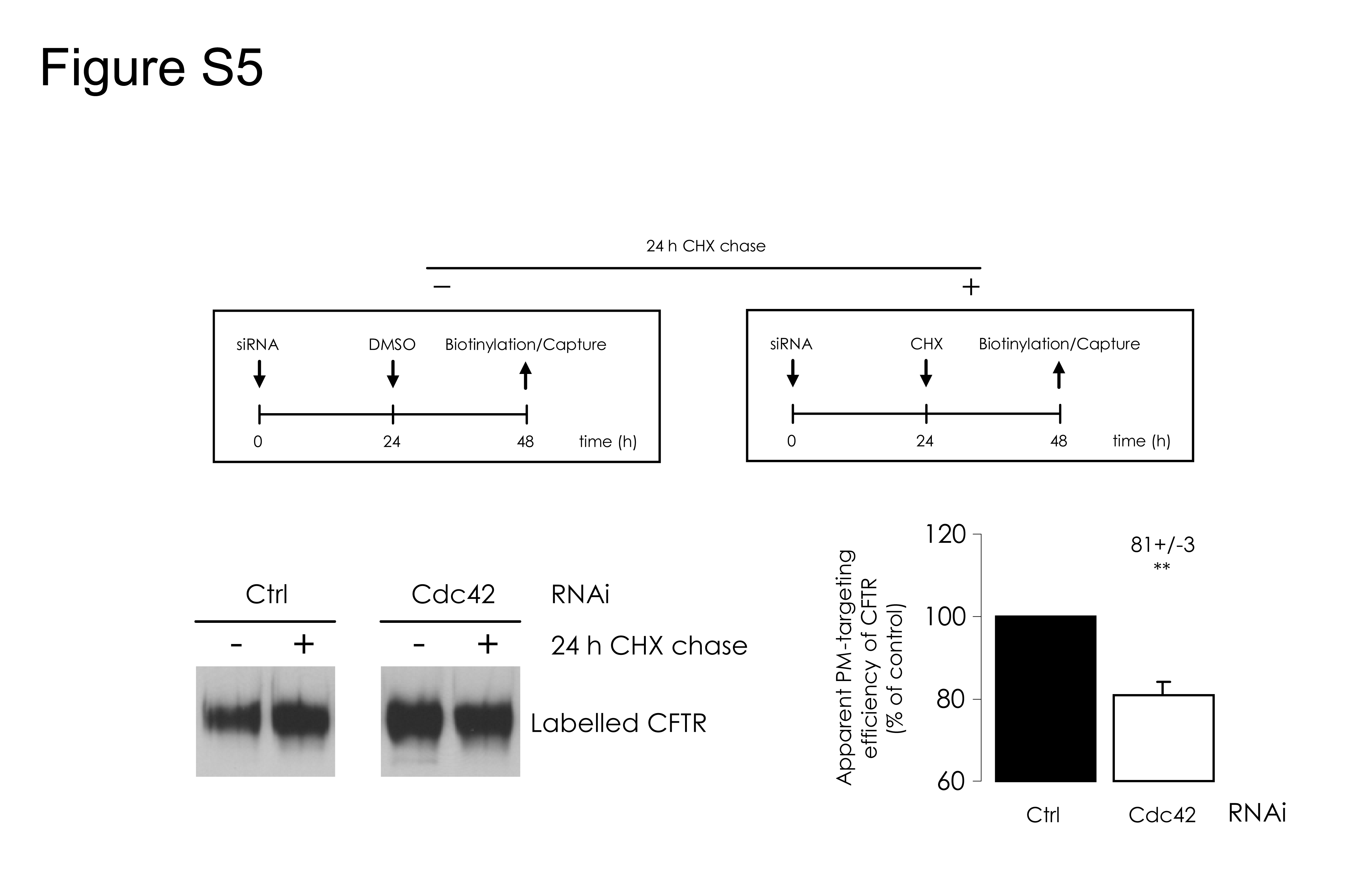

Supplement: S5 Fig — The upper diagrams summarize the procedure followed. Cells were siRNA-transfected to deplete Cdc42 protein and cultured for 48 h. In addition, cells were exposed to 100 μg/mL cycloheximide (+), or 0.1% DMSO (v/v) for the control condition (-), for the last 24 h. Afterwards, PM proteins were biotinylated and purified from 100 μg clarified lysates. Labelled CFTR protein amounts were assessed in the resulting samples by densitometric quantification of Western blot images (representative examples in the bottom left panel). In control RNAi condition, labelled CFTR amounts extracted from the same amount of whole cell lysates appeared higher after 24h CHX treatment: stability differences between the various cellular proteins may account for this paradox. We estimated PM-targeting efficiency by calculating (+) to (-) ratios, the Ctrl RNAi value being used as 100% of apparent PM-targeting efficiency. In the bottom right panel, histogram expresses the relative CFTR cell surface targeting efficiency. Data represent means ± SEM of 3 independent experiments, each performed in duplicate. **: p<0.01. (TIF) [file pone.0118943.s005.tif]
