# Supplementary material for: Involvement of the Cdc42 Pathway in CFTR Post-Translational Turnover and in Its Plasma Membrane Stability in Airway Epithelial Cells
Source: PLoS One. 2015 Mar 13;10(3):e0118943. doi: 10.1371/journal.pone.0118943 (PMC4359135; doi:10.1371/journal.pone.0118943)
Supplement: S1 Table — (DOC) [file pone.0118943.s006.doc]

| **Encoded protein** | **sense strand oligonucleotide sequence** |
| --- | --- |
| Cdc42 | 5'-CCGCUGAGUUAUCCACAAAdTdT-3' |
| N-WASP | 5'- CUUGUCAAGUUGCUCUUAAdTdT-3' |
| dynamin 2 | 5'-GCUAUGCCAUUAAGAACAUdTdT-3' |
| caveolin 1 | 5'-GCUUCCUGAUUGAGAUUCAdTdT-3' |
